# Supplementary material for: Molecular Screening Reveals De Novo Loss-of-Function NR4A2 Variants in Saudi Children with Autism Spectrum Disorders: A Single-Center Study
Source: Int J Mol Sci. 2025 Jun 7;26(12):5468. doi: 10.3390/ijms26125468 (PMC12193383; doi:10.3390/ijms26125468)
Supplement: Supplementary file 1 [file ijms-26-05468-s001.zip › ijms-3621070-supplementary.pdf]

Molecular Screening Reveals *De Novo* Loss-of-Function NR4A2 Variants in Saudi Children with Autism Spectrum Disorders: A Single-Center Study

Najwa M. Alharbi*, Wejdan F. Baaboud[1], Heba Shawky*, Aisha A. Alrofaidi, Reem M. Farsi, Khloud M. Algothmi, Shahira A. Hassoubah, Fatemah S. Basingab, Sheren A. Azhari, Mona G. Alharbi, Reham Yahya, Safiah Alhazmi

Table of Contents

Supplementary Figures

Fig S1: Electropherogram of proband A1……………………..2

Fig. S2: Electropherogram of five probands carrying a nonsense c.44_45insA (p.S16*) variant................3

Fig. S3: Electropherogram of proband A3……………………..4

Fig. S4: Electropherogram of proband A12…………………..5

Fig. S5: Determination of sample size via G-Power software..6

Supplementary Tables

Table S1: Distribution of intellectual scores among ASD cohort....………..............….................................7

Supplementary Figures

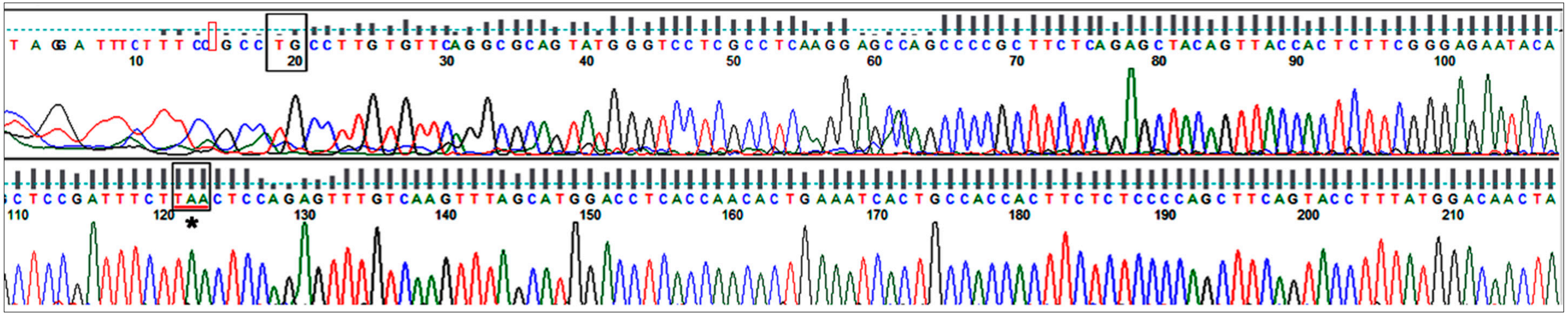

Figure S1. Electropherogram of proband A1 who carried multiple variants, including an indel (c.-2-2del) located in intron 2, concurrent with a start loss/nonsense c.1del (p.M1*), expected to trigger premature termination **(black asterisk)**. .

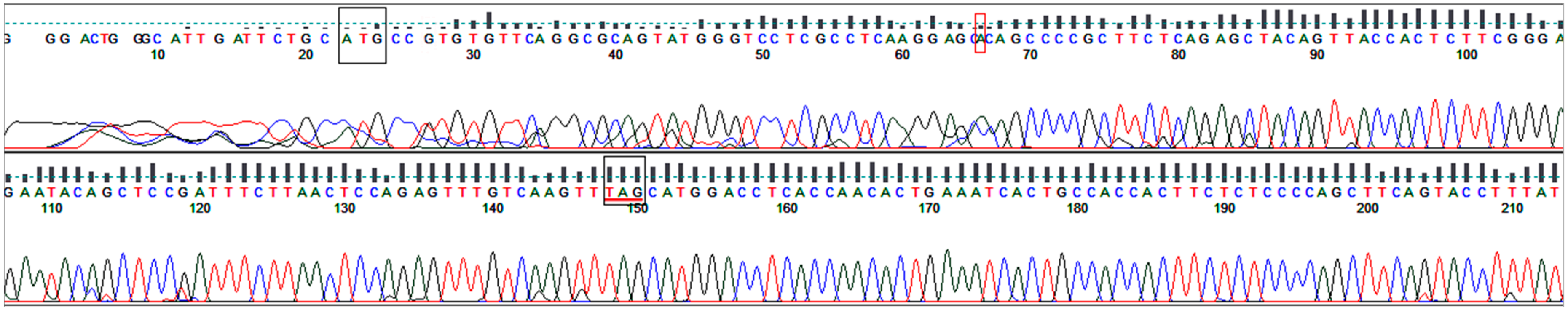

Figure S2. Electropherogram of five probands carrying a nonsense variant c.44_45insA (p.S16*) (boxed in red) that introduced an unexpected stop codon after 41 amino acids in the NTD region, concurrent with a frameshift/splice-acceptor loss CNV (c.1159--81_1540+67del, p.F387*) generated by a chromosomal deletion of 919 bp from the ligand-binding domain (LBD), encompassing exons 6 and 7.

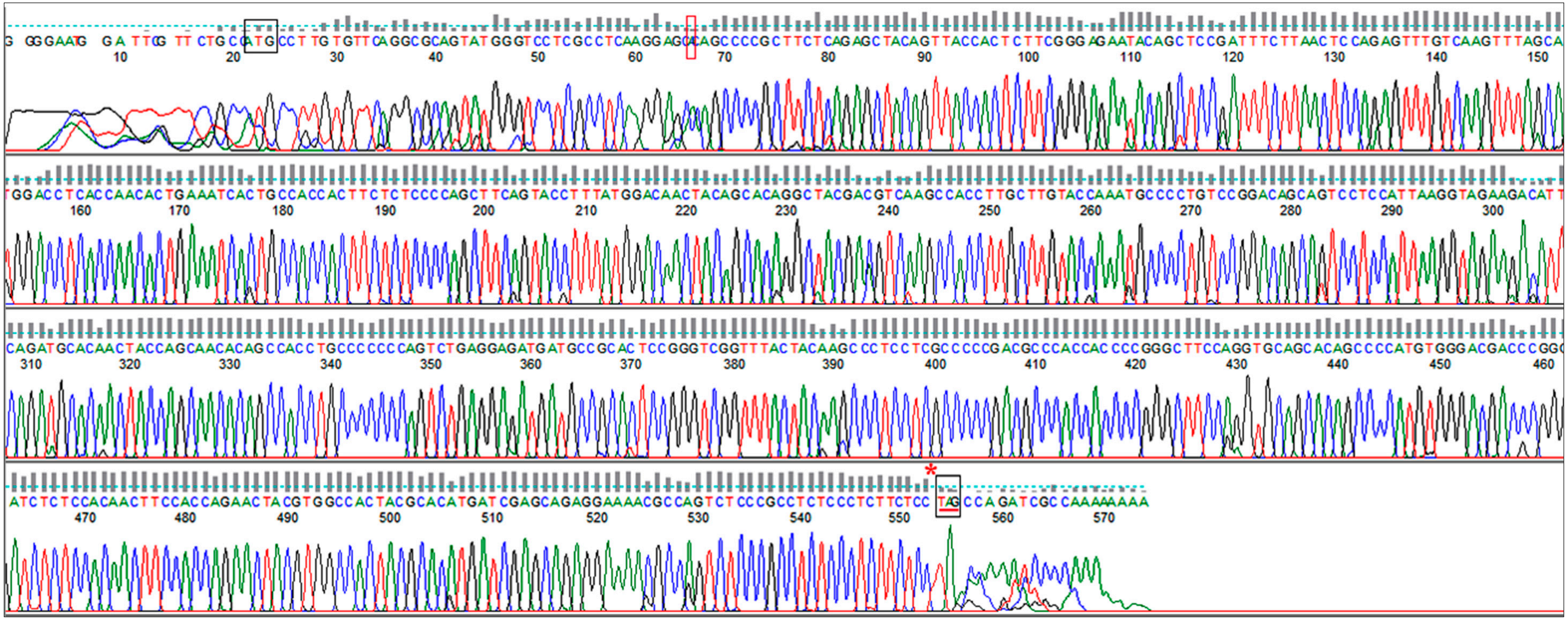

Figure S3. Electropherogram of proband A3 with a recurrent c.44_45insA variant (boxed in red), concurrent with a c.536del (p.K179*) nonsense variant expected to trigger premature termination **(red asterisk)**.

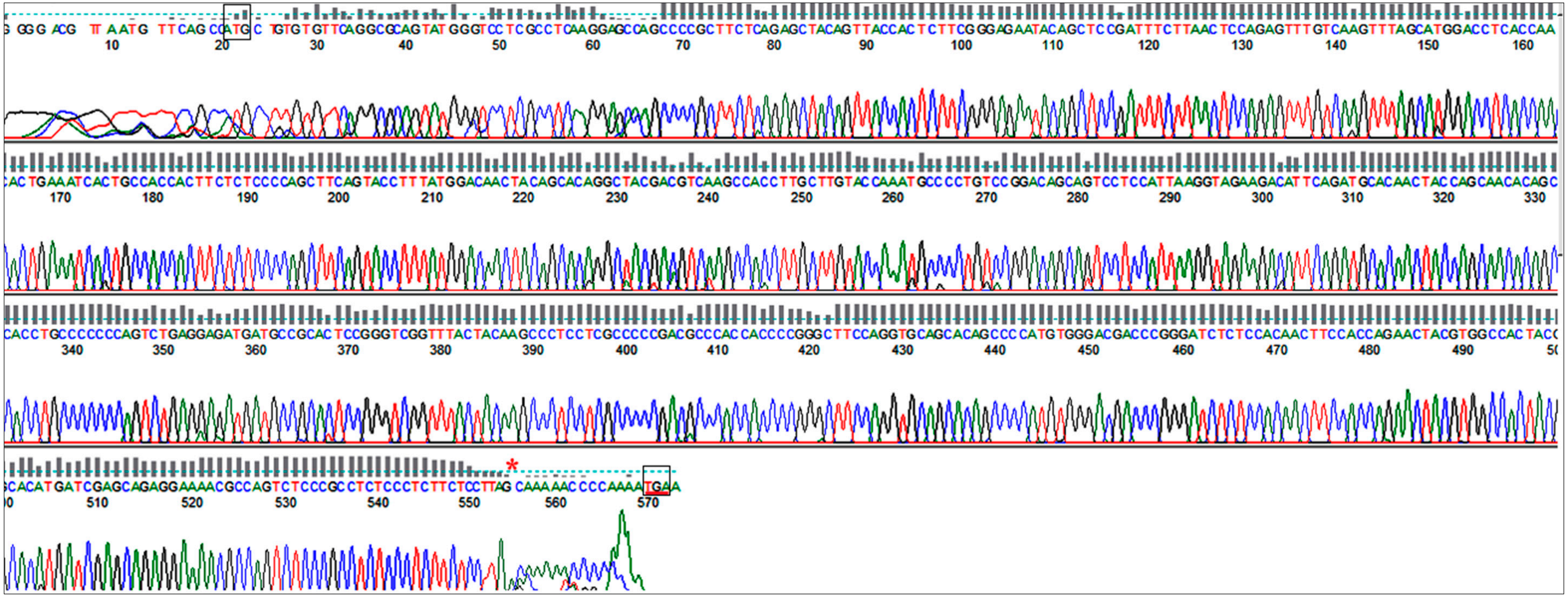

Figure S4. Electropherogram of proband A12 harboring a recurrent CNV, along with a nonsense variant (c.534del, p.F178*) which introduced an unexpected stop codon (**red asterisk)**.

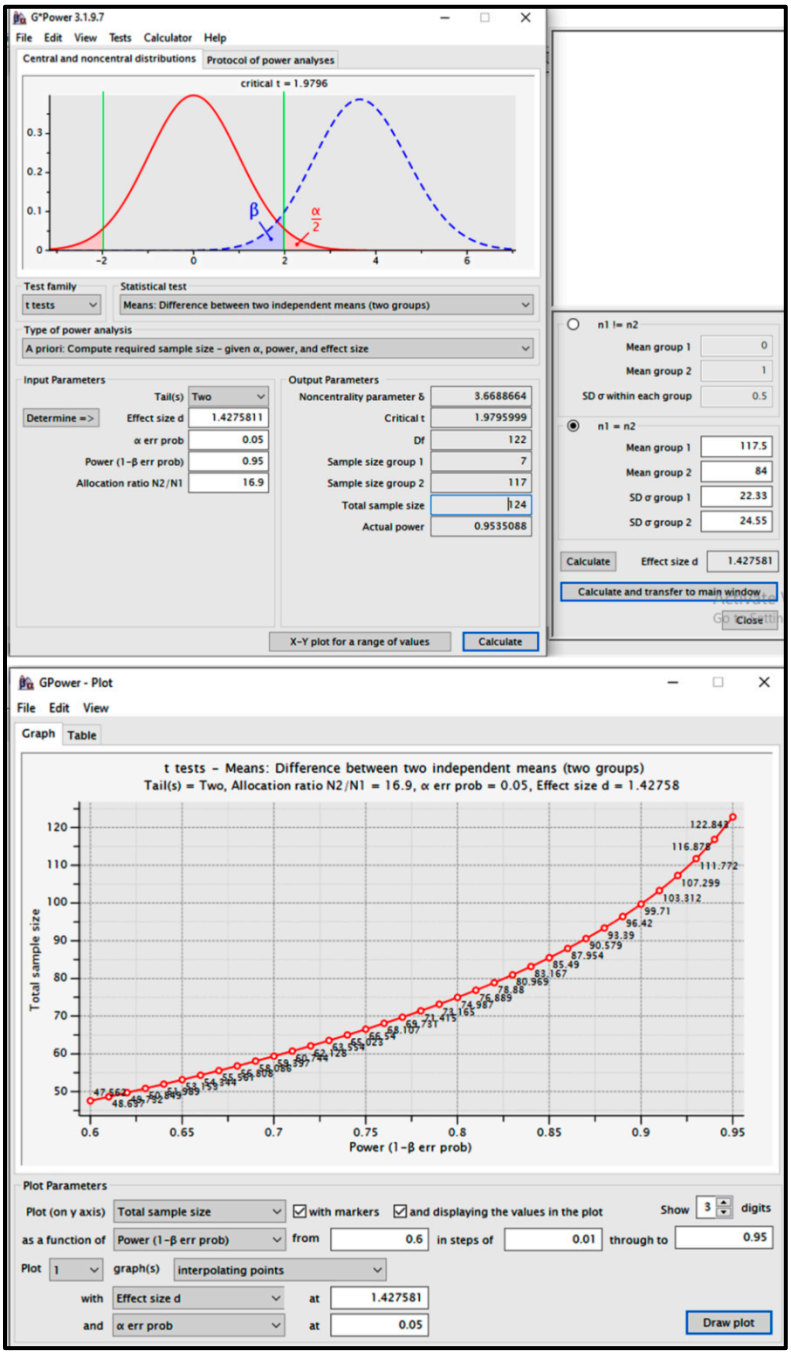

Figure S5. Determination of sample size via G-Power software. The analysis of two independent groups included in the study revealed a 1.427-fold difference in the FSIQ score in the ASD group relative to the discordant group (unaffected siblings), with average sample sizes of 17 and 7 for the ASD and discordant sibling groups, respectively, which were required to achieve an effect size (d) of 1.427 and a study power of 95% (1-β error probe).

Supplementary Tables

Table S1. Distribution of intellectual scores among ASD cohort

	SRS		FSIQ		VIQ		NVIQ		Vineland		PPVT	
	Mean ± SD (95% CI)	F, *P* value	Mean ± SD (95% CI)	F, *P* value	Mean ± SD (95% CI)	F, *P* value	Mean ± SD (95% CI)	F, *P* value	Mean ± SD (95% CI)	F, *P* value	Mean ± SD (95% CI)	F, *P* value
Age		1.395, 0.2574		4.173, 0.0216		5.051, 0.0098		3.313, 0.0446		0.67, 0.517		0.6189, 0.5426
3-5 (*n*= 74)	96.59 ± 7.383 (92.79 – 100.4)		106.1 ± 9.63 (100.9 – 111.2)		108.6 ± 11.85 (102.3 – 114.9)		105.9 ± 10.07 (100.5 – 111.2)		100.4 ± 12.37 (92.91 – 107.9)		90.12 ± 9.13 (85.42 – 94.81)	
6-9 (*n*= 125)	77.69 ± 4.468 (75.31 – 80.07)		81.54 ± 10.24 (75.35 – 87.73)		81.07 ± 12.88 (73.93 – 88.2)		90 ± 18.41 (79.37 – 100.6)		80.46 ± 16.38 (70.56 – 90.36)		77.56 ± 11.31 (71.53 – 83.59)	
10-14 (*n*= 139)	58.9 ± 15.7 (51.55 – 66.25)		59.15 ± 19.53 (50.01 – 68.29)		55.65 ± 21.4 (45.63 – 65.67)		74.57 ± 26.04 (63.31 – 85.82)		61.4 ± 17.76 (53.09 – 69.71)		62.2 ± 13.11 (56.06 – 68.34)	
**Gender**		1.408, 0.0598		1.098, 0.0566		1.211, 0.0184		1.2, 0.3441		1.101, 0.0242		1.1, 0.0537
Male (*n*= 244)	74.14 ± 19.15 (68.17 – 80.11)		78.38 ± 23.68 (71 – 85.76)		77 ± 25.62 (69.3 – 84.7)		86.48 ± 24.51 (78.84 – 94.11)		73.63 ± 21.39 (66.28 – 80.98)		73.6 ± 15.68 (68.71 – 78.48)	
Female (*n*= 94)	86.27 ± 16.14 (75.43 – 97.12)		94.18 ± 24.82 (77.51 – 110.9)		98.36 ± 28.19 (79.42 – 117.3)		94.27 ± 22.37 (79.24 – 109.3)		91.09 ± 22.44 (76.01 – 106.2)		84.18 ± 16.44 (73.14 – 95.22)	
**Total (*n*= 338)**	76.66 ± 19.08 (71.4 – 81.92)		81.66 ± 24.55 (74.89 – 88.43)		81.2 ± 27.26 (73.9 – 88.5)		88.09 ± 24.08 (81.46 – 94.73)		77.8 ± 22.68 (71.07 – 84.54)		75.79 ± 16.26 (71.31 – 80.28)	
Skewness	-0.8556		-0.5109		-0.3719		-0.6373		-0.364		-0.5142	

Abbreviations: SRS: social responsiveness scale; **FSIQ**: full-scale intelligence quotient; **VIQ**: verbal intelligence quotient; **NVIQ**: non-verbal intelligence quotient; **PPVT**: Peabody Picture Vocabulary Test

*All values are presented as the means ± SDs.
